# Editorial: Brain imaging for glycobiology

**DOI:** 10.3389/fnana.2022.1026499

**Published:** 2022-09-14

**Authors:** Takeshi Yoshimura, Satoru Yamagishi, Yoshihiro Akimoto, Sei Saitoh

**Affiliations:** ^1^Department of Child Development and Molecular Brain Science, United Graduate School of Child Development, Osaka University, Suita, Japan; ^2^Preeminent Medical Photonics Education & Research Center, Hamamatsu University School of Medicine, Hamamatsu, Japan; ^3^Department of Anatomy, Kyorin University School of Medicine, Mitaka, Japan; ^4^Department of Biomedical Molecular Sciences (Anatomy II), Fujita Health University School of Medicine, Toyoake, Japan

**Keywords:** brain imaging, glycomics, animal models, 3D volume imaging, brain imaging device, AI technology

The specific term “glycobiology” originated in 1988 and was proposed by Raymond Dwek (Rademacher et al., 1988). Glycosylation is the process by which a carbohydrate is covalently attached to a target macromolecule, which is typically a protein or lipid. The functions of glycosylation are diverse in various tissues, including the brain. As glycosylation is essential to brain development and function, glycobiology in the brain becomes an increasingly important theme. Glycans are essential for neurite outgrowth, axonal guidance, synaptogenesis, neural excitability, and neurotransmission by modulating the structure, stability, localization, and interaction profiles of proteins. The composition and abundance of glycans are changed by aging and diseases. Even as many investigators recognize the importance of glycans in the brain, our understanding of how brain glycan structure and function are correlated with their biological activities of brain glycans is still incomplete because of their structural diversity and differences in temporal and spatial abundance. Growing recognition of the structural complexity and functional importance of glycans and glycoconjugates in the brain has prompted intensive research in recent years. Therefore, imaging of glycans and glycoconjugates is essential to understand brain functioning. The aim of this Research Topic is to highlight the importance of imaging glycans and glycoconjugates in the brain, and to help readers appreciate their importance in the brain.

Hasan et al. feature mass spectrometry imaging (MSI) analysis of brain tissue glycans. MSI is a powerful tool used in mass spectrometry to visualize the spatial distribution of molecules, including glycans, in a variety of samples by their molecular masses. Difficulties posed by histological staining with lectins, antibodies, and chemical reporters can be circumvented by MSI. Studies of MSI have helped delineate the biological mechanisms and pathological characteristics of several brain diseases by visualization of glycans in brain specimens. MSI techniques are becoming increasingly valuable for glycobiology research.

Keratan sulfate (KS) is one of the glycosaminoglycans, a family of polysaccharides consisting of disaccharide unit repeats, and was first identified in corneal extracts by Suzuki (1939). Meyer and colleagues subsequently characterized this polysaccharide, renaming it kerato-sulfate (Meyer et al., 1953). In the human body, KS is most abundant in the cornea and brain. The significance of KS in brain development and certain pathological conditions has also been explored. Takeda-Uchimura et al. demonstrated that β1,3-*N*-acetylglucosaminyltransferase-7 (Beta3Gn-T7) is a major β1,3-*N*-acetylglucosaminyltransferase required for the synthesis of KS positive for the R-10G antibody in the adult mouse brain. Takeda-Uchimura et al. found that the mRNA encoding the Beta3Gn-T7 gene, B3gnt7, is selectively expressed in oligodendrocyte precursor cells and newly formed oligodendrocytes. Beta3Gn-T7 present in oligodendrocyte lineage cells could play a role in the formation of neuropils and perineuronal nets in the brain *via* synthesis of the R-10G-positive KS proteoglycan.

Heparan sulfate (HS) proteoglycans are comprised of a core protein with covalently linked long linear glycosaminoglycan HS chains. HS proteoglycans are ubiquitous on cell surfaces and in the extracellular matrix, including basement membranes. HS chains interact with a wide range of molecules. A Mini Review by Kerever and Arikawa-Hirasawa focuses on HS proteoglycans in the neurogenic niche, where new neurons are generated in the adult mammalian brain. HS proteoglycans are capable of regulating various growth factor signaling pathways that influence neurogenesis.

During development, when axons travel in the brain to find their target, they encounter various guidance cues. For example, the axon guidance cue netrin-1 was discovered in 1994 (Kennedy et al., 1994; Serafini et al., 1994). Draxin was relatively newly identified as an axon guidance cue in 2009 (Islam et al., 2009). A review by Ahmed and Shinmyo explains the multifaceted functions of the axon guidance cues netrin-1 and draxin in the development of neural circuits in the central nervous system. They also discuss the contribution of glycoproteins such as HS proteoglycans and Dystroglycan to the distribution and functions of netrin-1 and draxin.

Nio-Kobayashi and Itabashi focus on galectins and their ligand glycoconjugates in the brain. Galectins are carbohydrate binding proteins (lectins) that have a variety of roles in interacting with glycoproteins and glycolipids. Among the 15 members of the galectin family in mammals, only Galectin-1,−3,−4,−8, and −9 are predominantly expressed in the brain. Nio-Kobayashi and Itabashi summarize the expression patterns of each galectin in the brain and their functions in the regulation of neuronal and glial cell properties, as well as in the pathogenesis of neurodegenerative diseases.

A Mini Review by Itoh and Nishihara describes the crucial roles of mucin-type *O*-glycans in the *Drosophila* nervous system. Mucin-type *O*-glycans are important for central nervous system development, neuromuscular junction morphogenesis, and synaptic functions in *Drosophila*. Mucin-type *O*-glycans are evolutionarily conserved across species. The findings identifying a role for mucin-type *O*-glycans in the *Drosophila* nervous system will provide insight into the functions of this class of glycans in the mammalian brain.

In conclusion, the six articles presented in this Research Topic show the importance of imaging of glycans and glycoconjugates in the brain. We thank all contributors and readers, and hope that future work will elucidate roles of glycans and glycoconjugates in the brain, which are important both scientifically and clinically.

## Author contributions

TY, SY, YA, and SS designed the Research Topic. TY wrote the first draft of the manuscript. All authors contributed to manuscript revision, and read and approved the submitted version.

## Conflict of interest

The authors declare that the research was conducted in the absence of any commercial or financial relationships that could be construed as a potential conflict of interest.

## Publisher's note

All claims expressed in this article are solely those of the authors and do not necessarily represent those of their affiliated organizations, or those of the publisher, the editors and the reviewers. Any product that may be evaluated in this article, or claim that may be made by its manufacturer, is not guaranteed or endorsed by the publisher.
